# Association between urinary heavy metals, phthalates, phytoestrogens, and polycyclic aromatic hydrocarbons and endometriosis: A cross-sectional study from NHANES 1999 to 2016

**DOI:** 10.1097/MD.0000000000049300

**Published:** 2026-06-12

**Authors:** Yufeng Dong, Jing Qiu, Mengyun Yang, Wei Wang, Zhigang Tong

**Affiliations:** a Department of Gynaecology, Beilun People’s Hospital, Ningbo, Zhejiang, China; b Department of Nursing Teaching, Zhejiang Zhoushan Tourism and Health College, Zhoushan, Zhejiang, China; c Department of Gynaecology, Huzhou Maternity & Child Health Care Hospital, Huzhou, Zhejiang, China.

**Keywords:** BKMR, EM, PAHs, PE, phthalates, urine heavy metals

## Abstract

Research on the association between urinary heavy metals, phthalates, phytoestrogens (PEs), polycyclic aromatic hydrocarbons (PAHs), and endometriosis (EM) is limited. Data were from the National Health and Nutrition Examination Survey 1999 to 2016. Logistic regression models were used for analysis. In addition, qgcomp and Bayesian kernel machine regression models were employed to evaluate the effects of mixed exposures. After adjusting for covariates, compared with the first quartile (Q1), the fourth quartile (Q4) of urinary cobalt (95% confidence interval [CI] = 1.3–3.9) and urinary lead (95% CI = 1.0–3.1) were significantly associated with an increased risk of EM. Conversely, 1-naphthol (95% CI = 0.3–0.9), 2-fluorene (2-Flu; 95% CI = 0.3–0.9), 3-phenanthrene (95% CI = 0.3–1.0), and 2-phenanthrene (95% CI = 0.3–0.9) were negatively correlated with EM risk. When concentrations exceeded the 50th percentile, elevated levels of urinary heavy metal mixtures and PAH mixtures were positively associated with EM. In the Bayesian kernel machine regression mixture analysis, 2-Flu showed a positive association with EM, while 1-pyrene was negatively correlated, although the direction for 2-Flu differed from single-pollutant logistic regression. Analyses of chemical mixtures suggested possible associations for specific substances, including the PAH 2-Flu, the phthalate monobutyl phthalate, and the PEs enterodiol and enterolactone; however, the overall phthalate and PE mixtures were not significantly associated with EM, and these findings require further confirmation. Heavy metals (cobalt and lead) were consistently associated with increased EM risk.

## 1. Introduction

Endometriosis (EM) is defined as the presence of endometrial tissue outside the uterus. It is a chronic gynecological disease with a rising incidence, affecting 5% to 10% of women of childbearing age. Common symptoms include pelvic pain, infertility,^[1–3]^ dysmenorrhea, dyspareunia, psychiatric disorders, dysuria, and abdominal pain.^[4,5]^ EM has been linked to an increased risk of epithelial ovarian cancer,^[6]^ as well as gastrointestinal and immune disorders^[7]^; it is also associated with thyroid cancer. As an estrogen-dependent disease, EM can be influenced by genetics, environmental factors, and immune function.^[8,9]^

Evidence suggests associations between urinary heavy metals and environmental chemicals (ECs) – such as phthalates, phytoestrogens (PEs), and polycyclic aromatic hydrocarbons (PAHs) – and EM.^[10–12]^ Numerous epidemiological studies have shown that phthalate concentrations are typically higher in the bodily fluids of EM patients. Experimental studies indicate that phthalates can promote EM development by increasing proliferation markers and decreasing apoptosis markers.^[13]^ PEs are plant- and fungal-derived compounds that mimic estrogen but are nonsteroidal. Depending on various factors, they may exert both positive and negative effects on EM.^[14]^ For instance, genistein – a type of PE – has been shown to reduce the risk of EM by scavenging free radicals and downregulating the expression of stress response-related genes.^[15]^ Higher urinary levels of genistein and daidzein are associated with a decreased risk of advanced EM.^[16]^ High urinary concentrations of PAHs are linked to an increased risk of EM, with elevated 9-fluorene levels correlating with higher EM susceptibility in overweight participants.^[17]^ Heavy metals such as cadmium, copper, and cobalt are associated with EM primarily because they can interfere with hormone receptors or induce oxidative stress.^[18,19]^

A key analytical challenge in environmental epidemiology is disentangling the individual and combined effects of multiple, often highly correlated, chemical exposures. Traditional single-pollutant models may yield biased estimates in the presence of multicollinearity, necessitating mixture-based approaches such as Bayesian kernel machine regression (BKMR) and qgcomp. Recent interest has grown in exploring the connection between environmental exposures and EM, particularly focusing on the impact of urinary ECs such as heavy metals, phthalates, PEs, and PAHs. ECs are thought to contribute to EM pathogenesis by disrupting the endocrine system and affecting cell growth and death. A comprehensive investigation into the relationship between these ECs and EM is, therefore, crucial for understanding the disease’s pathogenesis and implementing effective prevention and control strategies. This study aimed to analyze the association between urinary levels of heavy metals, phthalates, PEs, and PAHs and the risk of EM. Using data from the National Health and Nutrition Examination Survey (NHANES) spanning 1999 to 2016, we sought to determine the relative significance of these pollutants within the overall exposure mixture.

## 2. Materials and methods

### 2.1. Study population

The NHANES conducted from 1999 to 2016 included a total of 41,474 participants. Initially, 20,264 male participants were excluded, followed by the exclusion of 15,653 participants with missing EM outcome data. Ultimately, 5557 participants were included in the analysis. NHANES is a cross-sectional survey that employs a multistage probability sampling design to collect demographic, clinical, behavioral, dietary, social, and laboratory data on the health and nutritional status of noninstitutionalized populations in the United States. The NHANES protocol was approved by the National Center for Health Statistics Institutional Review Board, and written informed consent was obtained from all participants.

### 2.2. Measurement of environmental mixtures

The following urinary analytes were determined: 11 heavy metals (barium [Ba], beryllium [Be], cadmium [Cd], cobalt [Co], cesium [Cs], molybdenum [Mo], lead [Pb], platinum [Pt], antimony [Sb], thallium [Tl], tungsten [W]); 7 phthalates (monobutyl phthalate [MBP], monocyclohexyl phthalate [MCP], monoethyl phthalate [MEP], mono-(2-ethylhexyl) phthalate [MHP], monoisononyl phthalate [MNP], monooctyl phthalate [MOP], monobenzyl phthalate [MZP]); 6 PEs (daidzein [DAZ], O-desmethylangolensin [DMA], equol [EQU], enterodiol [ETD], enterolactone [ETL], genistein [GNS]); 8 PAHs (1-naphthol [1-Nap], 2-naphthol [2-Nap], 3-fluorene [3-Flu], 2-fluorene [2-Flu], 3-phenanthrene [3-PA], 1-phenanthrene [1-PA], 2-phenanthrene [2-PA], 1-pyrene [1-Pyr]); as well as *Chlamydia trachomatis* and gonorrhea.

### 2.3. EM definitions

Data were collected via questionnaires. Participants were classified as having EM based on their response to the question: “Have you ever been told by a doctor or other health professional that you have endometriosis?” Those who answered “yes” were categorized as EM cases.

### 2.4. Covariates

Based on previous studies, covariates potentially associated with urinary metal exposure and EM prevalence were selected, including age, race/ethnicity, educational level, marital status, family size, and household income.

### 2.5. Statistical methods

Descriptive statistics were used to summarize the demographic characteristics of participants and the concentrations of urinary ECs, including heavy metals, phthalates, phenols, and PAHs. Non-normally distributed continuous variables were presented as median and interquartile range, with group comparisons performed using the Mann–Whitney *U* test. Categorical variables were reported as frequencies, and group comparisons were conducted using the chi-square (χ^2^) test. To account for variations in urinary dilution, urinary creatinine concentration was included as a covariate in all regression models. Prior to statistical analysis, urinary ECs were subjected to natural log transformation (ln transformation) to achieve a normal distribution. Spearman correlation was used to assess relationships between chemicals, and ECs were also transformed into quartiles. Logistic regression models and weighted logistic regression were employed to examine the associations between ln-transformed and quartile-transformed concentrations of each environmental compound and EM.

A quantile-based approach – specifically quantile g-computation (qgcomp) and BKMR – was used to explore the relationship between urinary ECs and EM. The qgcomp model assessed the combined effect of different quartiles of urinary EC concentrations without assuming directional homogeneity. The BKMR model uses a kernel-based framework that inherently accommodates multicollinearity by modeling the joint exposure–response surface without requiring independent predictors. The qgcomp approach employs quantile transformation and bootstrap resampling, making it robust to correlations among exposures. This method integrates the simplicity of weighted quantile sum regression (WQS) with the flexibility of g-computation to determine the weights of each EC, thereby evaluating their relative importance within the mixture. The model used a binomial distribution as the link function, adopted a quadratic model, and applied a 500-time bootstrap method to calculate confidence intervals (CIs) and *P* values. In addition, the BKMR model estimated the exposure–response relationship between urinary ECs and EM. This semi-parametric approach accommodates nonlinear and nonadditive relationships and calculates the inclusion probability of each EC to assess the relative importance of different chemicals in the EC mixture.

All analyses were performed using R software (version 4.3.1; R Foundation for Statistical Computing, Vienna, Austria). The “survey,” “qgcomp,” and “bkmr” packages were used for weighted logistic regression, qgcomp, and BKMR analyses, respectively. All tests were two-sided, with a significance level set at 0.05.

## 3. Results

### 3.1. Baseline characteristics of participants

After excluding male participants (n = 41,474) and those with missing EM outcome data (n = 15,653), a total of 5557 participants were included in this study. Among them, 380 had EM, corresponding to a prevalence of 6.94%. Significant differences in prevalence were observed based on age, ethnicity, education level, marital status, family size, household income, as well as levels of Co, ETL, 1-Nap, 2-Flu, 1-PA, 2-PA, and 3-PA (Table 1).

**Table 1:** Characteristics of participants classified by endometriosis.

	Endometriosis		Statistic	*P*
	Yes	No		
To all	380	5177		
Age	40.00 (34.00–47.00)	34.00 (26.00–44.00)	9.038	<.001
Race			97.271	<.001
Mexican American	30	1284		
Other Hispanic	7	264		
Non-Hispanic White	261	2307		
Non-Hispanic Black	67	1084		
Other race	15	238		
Education			35.646	<.001
<9th grade	7	468		
9–11th grade	41	828		
High school grad/GED or equivalent	101	1142		
Some college or AA degree	133	1616		
College graduate or above	98	1118		
Marital			25.192	<.001
Married	230	2757		
Divorced	83	892		
Never married	67	1528		
Family population			29.486	<.001
≤2	150	1522		
3–5	209	2958		
>5	21	697		
Family income			28.240	<.001
<20,000	55	989		
20,000–64,999	127	1925		
≥65,000	184	1861		
Urinary gonorrhea			0.430	.512
Yes	0	8		
No	171	3180		
Urinary chlamydia			0.627	.428
Yes	2	65		
No	169	3123		
Metals – urine				
Ba	1.29 (0.61–2.75)	1.46 (0.73–2.89)	1.258	.209
Be	0.08 (0.05–0.09)	0.08 (0.05–0.09)	0.743	.458
Cd	0.32 (0.13–0.53)	0.25 (0.12–0.50)	1.003	.316
Co	0.36 (0.21–0.65)	0.46 (0.26–0.76)	2.935	.003
Cs	4.60 (2.44–8.31)	5.02 (2.94–7.42)	0.752	.452
Mo	38.95 (21.15–66.28)	45.80 (23.50–75.30)	1.603	.109
Pb	0.58 (0.23–0.99)	0.60 (0.34–1.10)	1.870	.061
Pt	0.03 (0.01–0.05)	0.03 (0.01–0.03)	0.517	.605
Sb	0.10 (0.05–0.17)	0.10 (0.05–0.16)	0.258	.796
Tl	0.16 (0.08–0.27)	0.18 (0.11–0.27)	1.640	.101
W	0.08 (0.04–0.14)	0.07 (0.03–0.14)	0.949	.342
Phthalates – urine				
MBP	24.40 (11.10–41.03)	24.20 (10.90–46.30)	0.134	.894
MCP	0.43 (0.43–1.28)	0.43 (0.43–1.28)	0.029	.977
MEP	119.76 (47.55–303.06)	154.31 (58.28–383.20)	1.889	.059
MHP	2.30 (0.81–6.70)	3.40 (0.85–8.10)	1.948	.051
MNP	0.87 (0.87–1.09)	0.87 (0.87–1.09)	1.156	.248
MOP	1.19 (1.19–1.31)	1.19 (1.19–1.31)	0.327	.744
MZP	10.76 (3.92–23.54)	10.01 (4.03–20.81)	0.125	.901
Phytoestrogens – urine				
DAZ	43.63 (14.25–155.50)	51.65 (17.70–180.65)	0.551	.582
DMA	2.89 (0.53–21.21)	2.90 (0.50–17.65)	0.542	.588
EQU	6.20 (2.33–18.10)	7.30 (2.33–15.60)	0.280	.779
ETD	39.45 (14.58–88.80)	38.44 (14.40–97.08)	0.434	.664
ETL	283.50 (70.03–729.92)	342.00 (112.00–780.00)	2.036	.042
GNS	23.72 (5.60–107.34)	23.80 (8.70–74.98)	0.029	.977
PAHs – urine				
1-Nap	3165.85 (1027.73–11,525.28)	1635.65 (747.53–5302.55)	2.892	.004
2-Nap	4717.70 (1831.90–13,548.45)	3306.25 (1506.40–8502.30)	1.852	.064
3-Flu	134.00 (49.95–612.75)	83.50 (41.10–227.00)	2.414	.016
2-Flu	343.00 (178.50–1159.25)	241.30 (123.00–554.30)	2.714	.007
3-PA	104.45 (54.65–213.85)	86.35 (45.00–171.00)	2.219	.026
1-PA	188.15 (93.03–392.05)	148.00 (80.05–284.25)	2.046	.041
2-PA	74.00 (39.00–162.60)	58.95 (29.08–123.15)	2.200	.028
1-Pyr	78.10 (35.10–183.15)	71.40 (33.90–156.40)	0.991	.322

1-Nap = 1-hydroxynaphthalene, 1-PA = 1-hydroxyphenanthrene, 1-Pyr = 1-hydroxypyrene, 2-Flu = 2-hydroxyfluorene, 2-Nap = 2-hydroxynaphthalene, 2-PA = 2-hydroxyphenanthrene, 3-Flu = 3-hydroxyfluorene, 3-PA = 3-hydroxyphenanthrene, Ba = barium, Be = beryllium, Cd = cadmium, Co = cobalt, Cs = cesium, DAZ = daidzein, DMA = O-desmethylangolensin, ECs = environmental chemicals, EQU = equol, ETD = enterodiol, ETL = enterolactone, GNS = genistein, MBP = mono-n-butyl phthalate, MCP = monocyclohexyl phthalate, MEP = monoethyl phthalate, MHP = mono-(2-ethyl)-hexyl phthalate, MNP = monoisononyl phthalate, Mo = molybdenum, MOP = mono-n-octyl phthalate, MZP = monobenzyl phthalate, PAHs = polycyclic aromatic hydrocarbons, Pb = lead, Pt = platinum, Sb = antimony, Tl = thallium, W = tungsten.

### 3.2. Logistic regression of urine ECs concentration and health outcomes

Univariate logistic regression analysis was performed to examine the relationship between urinary chemical concentrations and EM. The results showed that higher urinary levels of Pb (OR = 1.39, 95% CI = 1.01–1.90, *P* = .044), ETL (OR = 1.00, 95% CI = 1.00–1.00, *P* = .038), 2-Nap (OR = 1.00, 95% CI = 1.00–1.00, *P* = .023), 2-Flu (OR = 1.00, 95% CI = 1.00–1.00, *P* = .020), and 3-Flu (OR = 1.00, 95% CI = 1.00–1.00, *P* = .002) were associated with an increased risk of EM, with all differences reaching statistical significance (*P* < .05; Table 2).

**Table 2:** Univariate logistic regression results of chemical concentrations in urine and endometriosis.

Variables	β	SD	Wald	OR (95% CI)	*P*
Metals – urine					
Ba	0.054	0.042	1.690	1.06 (0.97–1.15)	.194
Be	2.237	5.307	0.178	9.37 (<0.01–308,496.37)	.673
Cd	−0.04	0.061	0.433	0.96 (0.85–1.08)	.511
Co	0.065	0.143	0.206	1.07 (0.81–1.41)	.650
Cs	−0.001	0.009	0.004	1.00 (0.98–1.02)	.947
Mo	0.004	0.002	2.768	1.00 (1.00–1.01)	.096
Pb	0.326	0.162	4.040	1.39 (1.01–1.90)	.044
Pt	−2.937	1.881	2.438	0.05 (0.00–2.12)	.118
Sb	0.215	0.631	0.116	1.24 (0.36–4.27)	.734
Tl	1.308	0.788	2.754	3.70 (0.79–17.34)	.097
W	0.081	0.356	0.051	1.08 (0.54–2.18)	.821
Phthalates – urine					
MBP	−0.001	0.001	2.112	1.00 (1.00–1.00)	.146
MCP	0.112	0.161	0.487	1.12 (0.82–1.53)	.485
MEP	0	0	0.307	1.00 (1.00–1.00)	.580
MHP	0.004	0.005	0.756	1.00 (1.00–1.01)	.385
MNP	0.110	0.096	1.301	1.12 (0.92–1.35)	.254
MOP	0.097	0.138	0.495	1.10 (0.84–1.45)	.482
MZP	0.003	0.003	0.908	1.00 (1.00–1.01)	.341
Phytoestrogens – urine					
DAZ	0	0	2.214	1.00 (1.00–1.00)	.137
DMA	0	0	0.157	1.00 (1.00–1.00)	.692
EQU	0	0	0.020	1.00 (1.00–1.00)	.889
ETD	0	0.001	0.640	1.00 (1.00–1.00)	.424
ETL	0	0	4.291	1.00 (1.00–1.00)	.038
GNS	0	0	3.347	1.00 (1.00–1.00)	.067
PAHs – urine					
1-Nap	0	0	2.201	1.00 (1.00–1.00)	.138
2-Nap	0	0	5.198	1.00 (1.00–1.00)	.023
3-Flu	0	0	9.141	1.00 (1.00–1.00)	.002
2-Flu	0	0	5.372	1.00 (1.00–1.00)	.020
3-PA	0	0	0.259	1.00 (1.00–1.00)	.611
1-PA	0	0	1.304	1.00 (1.00–1.00)	.253
2-PA	0	0	0.585	1.00 (1.00–1.00)	.444
1-Pyr	0	0	1.033	1.00 (1.00–1.00)	.309

1-Nap = 1-hydroxynaphthalene, 1-PA = 1-hydroxyphenanthrene, 1-Pyr = 1-hydroxypyrene, 2-Flu = 2-hydroxyfluorene, 2-Nap = 2-hydroxynaphthalene, 2-PA = 2-hydroxyphenanthrene, 3-Flu = 3-hydroxyfluorene, 3-PA = 3-hydroxyphenanthrene, Ba = barium, Be = beryllium, Cd = cadmium, Co = cobalt, Cs = cesium, DAZ = daidzein, DMA = O-desmethylangolensin, ECs = environmental chemicals, EQU = equol, ETD = enterodiol, ETL = enterolactone, GNS = genistein, MBP = mono-n-butyl phthalate, MCP = monocyclohexyl phthalate, MEP = monoethyl phthalate, MHP = mono-(2-ethyl)-hexyl phthalate, MNP = monoisononyl phthalate, Mo = molybdenum, MOP = mono-n-octyl phthalate, MZP = monobenzyl phthalate, PAHs = polycyclic aromatic hydrocarbons, Pb = lead, Pt = platinum, Sb = antimony, Tl = thallium, W = tungsten.

For ln-transformed continuous exposures, the odds ratios in Table 2 represent the effect per 1-unit increase on the natural log scale. Given the typically small interquartile ranges of these urinary biomarkers, a 1-unit increase corresponds to a very modest absolute change, yielding ORs that round to 1.00 (95% CI = 1.00–1.00). Despite the rounding, the *P* values confirm statistically significant associations for these compounds.

After adjusting for covariates (age, ethnicity, education level, marital status, family size, and household income), weighted logistic regression analyses were conducted on quartile-transformed (natural log-transformed) urinary levels of heavy metals, phthalates, PEs, and PAHs. Quartile 1 (Q1) served as the reference group, with comparisons made for Q2, Q3, and Q4. Compared with Q1, Q4 levels of Co (OR = 2.2, 95% CI = 1.3–3.9) and Pb (OR = 1.8, 95% CI = 1.0–3.1) were significantly positively associated with EM risk. Conversely, Q4 levels of 1-Nap (OR = 0.5, 95% CI = 0.3–0.9), 2-Flu (OR = 0.5, 95% CI = 0.3–0.9), 3-PA (OR = 0.5, 95% CI = 0.3–1.0), and 2-PA (OR = 0.5, 95% CI = 0.3–0.9) were significantly negatively associated with EM risk (Table 3).

**Table 3:** Weighted logistic regression analysis.

ECs	Quartile 1	Quartile 2		Quartile 3		Quartile 4	
		OR (95% CI)	*P*	OR (95% CI)	*P*	OR (95% CI)	*P*
Metals – urine							
BA	Ref	1.1 (0.7–1.9)	.695	1.3 (0.8–2.2)	.346	1.3 (0.8–2.2)	.342
CD	Ref	1.0 (0.6–1.8)	.987	0.6 (0.4–1.0)	.069	0.8 (0.5–1.4)	.484
CO	Ref	1.5 (0.9–2.4)	.129	1.5 (0.9–2.6)	.089	2.2 (1.3–3.9)	.005
CS	Ref	1.4 (0.8–2.3)	.230	1.9 (1.1–3.3)	.026	1.1 (0.7–1.7)	.811
MO	Ref	1.3 (0.8–2.2)	.330	1.1 (0.7–1.8)	.776	1.6 (0.9–2.8)	.096
PB	Ref	1.2 (0.7–2.1)	.453	1.3 (0.8–2.1)	.316	1.8 (1.0–3.1)	.041
SB	Ref	1.3 (0.8–2.3)	.301	1.4 (0.8–2.3)	.226	1.1 (0.7–1.8)	.701
TL	Ref	1.0 (0.6–1.6)	.995	2.0 (1.1–3.6)	.018	1.3 (0.8–2.2)	.310
W	Ref	0.7 (0.3–1.4)	.315	0.6 (0.3–1.3)	.217	0.7 (0.3–1.5)	.379
Phthalates and phytoestrogens – urine							
MBP	Ref	0.9 (0.6–1.6)	.789	0.9 (0.5–1.5)	.616	1.1 (0.6–1.8)	.796
MEP	Ref	1.0 (0.6–1.5)	.924	2.2 (1.3–3.9)	.006	1.5 (0.9–2.4)	.132
MZP	Ref	1.3 (0.8–2.2)	.354	1.1 (0.6–1.8)	.805	0.9 (0.6–1.5)	.746
DAZ	Ref	1.5 (0.9–2.4)	.135	1.3 (0.8–2.1)	.276	1.4 (0.9–2.3)	.176
DMA	Ref	0.9 (0.5–1.5)	.677	0.9 (0.6–1.6)	.824	0.9 (0.6–1.6)	.824
EQU	Ref	0.7 (0.4–1.2)	.173	1.1 (0.6–2.1)	.698	0.7 (0.4–1.3)	.289
ETD	Ref	1.0 (0.6–1.7)	.917	0.9 (0.5–1.5)	.683	1.1 (0.6–1.8)	.806
ETL	Ref	1.1 (0.7–1.8)	.621	1.7 (1.0–2.9)	.049	1.3 (0.8–2.2)	.265
GNS	Ref	2.1 (1.2–3.5)	.008	1.5 (0.9–2.5)	.085	1.2 (0.8–1.9)	.454
PAHs – urine							
1-Nap	Ref	1.1 (0.5–2.1)	.855	0.7 (0.4–1.4)	.346	0.5 (0.3–0.9)	.031
2-Nap	Ref	1.3 (0.6–2.5)	.488	0.7 (0.4–1.2)	.191	0.7 (0.4–1.3)	.233
3-Flu	Ref	1.5 (0.8–2.9)	.254	1.1 (0.6–2.0)	.867	0.6 (0.3–1.0)	.072
2-Flu	Ref	0.7 (0.3–1.3)	.239	0.6 (0.3–1.1)	.080	0.5 (0.3–0.9)	.023
3-PA	Ref	0.7 (0.4–1.3)	.243	0.7 (0.4–1.3)	.247	0.5 (0.3–1.0)	.034
1-PA	Ref	1.0 (0.5–1.9)	.961	0.9 (0.5–1.8)	.841	0.6 (0.3–1.1)	.107
2-PA	Ref	0.6 (0.3–1.2)	.184	0.6 (0.3–1.2)	.170	0.5 (0.3–0.9)	.028
1-Pyr	Ref	1.1 (0.6–2.0)	.747	0.9 (0.5–1.7)	.773	1.0 (0.5–1.7)	.880

1-Nap = 1-hydroxynaphthalene, 1-PA = 1-hydroxyphenanthrene, 1-Pyr = 1-hydroxypyrene, 2-Flu = 2-hydroxyfluorene, 2-Nap = 2-hydroxynaphthalene, 2-PA = 2-hydroxyphenanthrene, 3-Flu = 3-hydroxyfluorene, 3-PA = 3-hydroxyphenanthrene, Ba = barium, Be = beryllium, Cd = cadmium, Co = cobalt, Cs = cesium, DAZ = daidzein, DMA = O-desmethylangolensin, ECs = environmental chemicals, EQU = equol, ETD = enterodiol, ETL = enterolactone, GNS = genistein, MBP = mono-n-butyl phthalate, MCP = monocyclohexyl phthalate, MEP = monoethyl phthalate, MHP = mono-(2-ethyl)-hexyl phthalate, MNP = monoisononyl phthalate, Mo = molybdenum, MOP = mono-n-octyl phthalate, MZP = monobenzyl phthalate, PAHs = polycyclic aromatic hydrocarbons, Pb = lead, Pt = platinum, Sb = antimony, Tl = thallium, W = tungsten.

### 3.3. Correlation analysis between the concentrations of urine ECs

**Figure 1.**
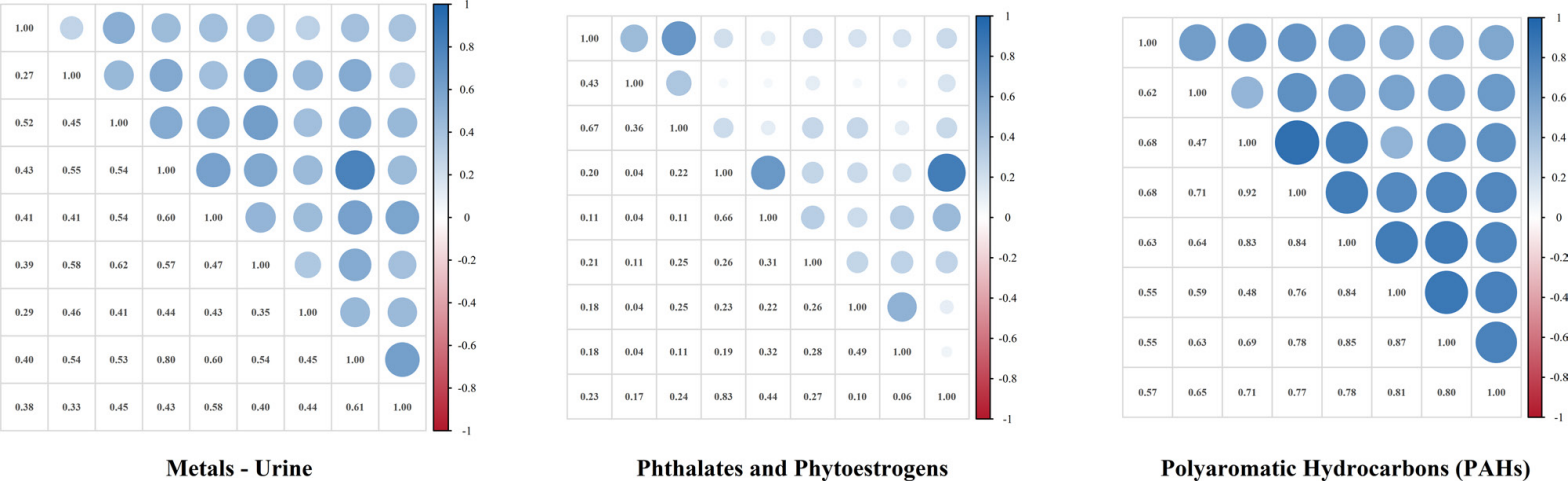
Pearson correlation between urine environmental chemicals. Significant correlations were identified between the concentrations of 9 chemicals in urine heavy metals and 8 chemicals in PAHs. Statistical analysis revealed strong correlations (*P* < .001). Urine ECs underwent natural log transformation. 1-Nap = 1-hydroxynaphthalene, 1-PA = 1-hydroxyphenanthrene, 1-Pyr = 1-hydroxypyrene, 2-Flu = 2-hydroxyfluorene, 2-Nap = 2-hydroxynaphthalene, 2-PA = 2-hydroxyphenanthrene, 3-Flu = 3-hydroxyfluorene, 3-PA = 3-hydroxyphenanthrene, Ba = barium, Cd = cadmium, Co = cobalt, Cs = cesium, DAZ = daidzein, DMA = O-desmethylangolensin, ECs = environmental chemicals, EQU = equol, ETD = enterodiol, ETL = enterolactone, GNS = genistein, MBP = mono-n-butyl phthalate, MEP = monoethyl phthalate, Mo = molybdenum, MZP = monobenzyl phthalate, PAHs = polycyclic aromatic hydrocarbons, Pb = lead, Sb = antimony, Tl = thallium, W = tungsten.

Significant correlations were identified between concentrations of 9 urinary heavy metals and 8 urinary PAHs. Statistical analysis revealed strong correlations (*P* < .001) with correlation coefficients ranging from 0.27 to 0.80 (for heavy metals) and 0.55 to 0.92 (for PAHs), respectively. Notably, in correlation analyses between 9 chemicals and those in PEs, significant correlations were observed between MBP and MZP (*r* = 0.67), DAZ and DMA (*r* = 0.66), and DAZ and GNS (*r* = 0.83; Fig. 1).

### 3.4. Bayesian kernel machine regression

**Figure 2.**
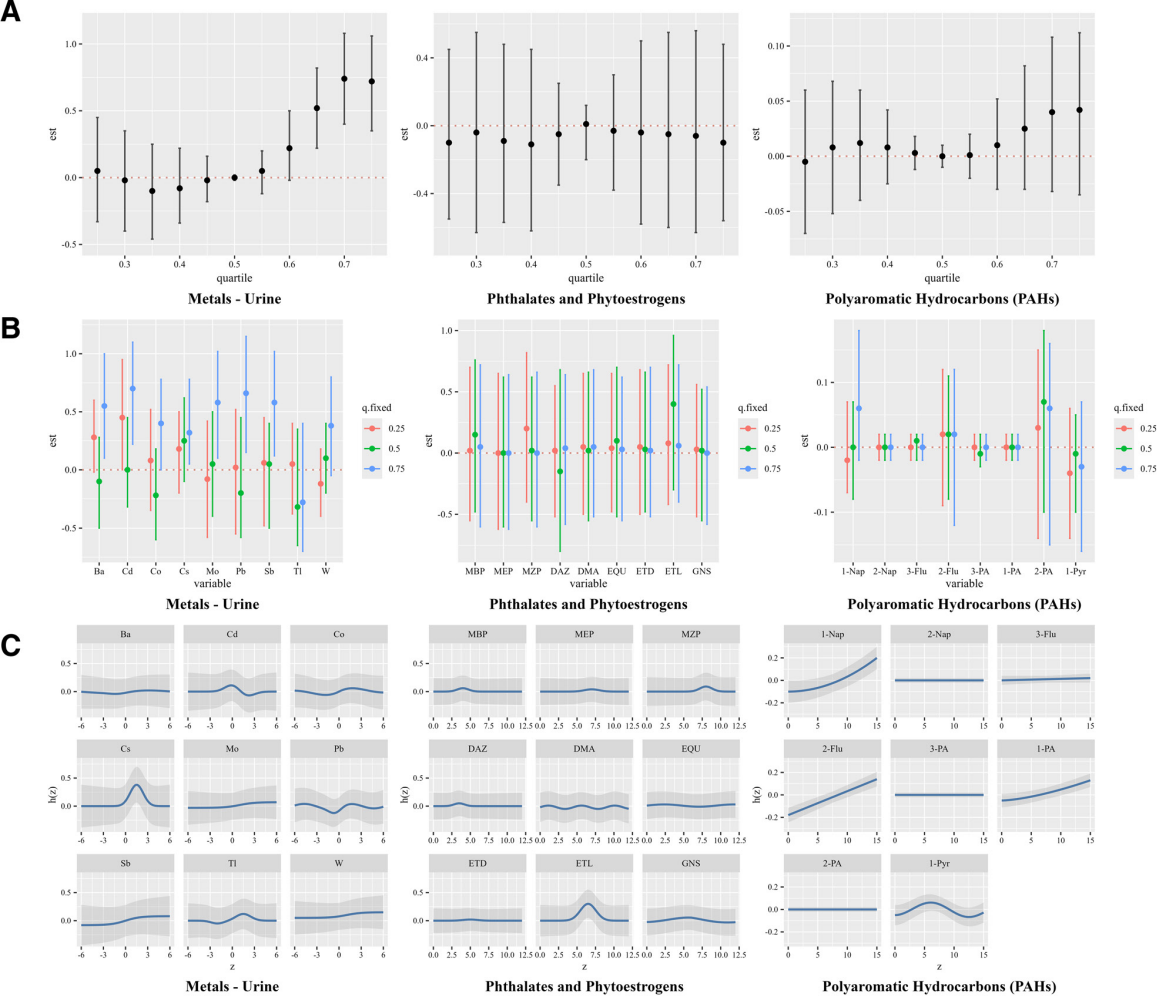
The association between urine environmental chemicals and endometriosis was analyzed using the BKMR model. (A) Overall association between urine environmental chemicals and endometriosis. (B) Univariate effects of urine environmental chemicals. (C) Univariate exposure–response relationship for the concentration of each substance in urine environmental chemicals. 1-Nap = 1-hydroxynaphthalene, 1-PA = 1-hydroxyphenanthrene, 1-Pyr = 1-hydroxypyrene, 2-Flu = 2-hydroxyfluorene, 2-Nap = 2-hydroxynaphthalene, 2-PA = 2-hydroxyphenanthrene, 3-Flu = 3-hydroxyfluorene, 3-PA = 3-hydroxyphenanthrene, Ba = barium, BKMR = Bayesian kernel machine regression, Cd = cadmium, Co = cobalt, Cs = cesium, DAZ = daidzein, DMA = O-desmethylangolensin, ECs = environmental chemicals, EQU = equol, ETD = enterodiol, ETL = enterolactone, GNS = genistein, MBP = mono-n-butyl phthalate, MEP = monoethyl phthalate, Mo = molybdenum, MZP = monobenzyl phthalate, PAHs = polycyclic aromatic hydrocarbons, Pb = lead, Sb = antimony, Tl = thallium, W = tungsten.

The BKMR model was used to investigate the association between cumulative exposure to urinary ECs and EM. Results from multi-pollutant modeling indicated that mixtures of urinary heavy metals and PAHs were positively associated with EM when their concentrations exceeded the 50th percentile. However, changes in phthalate and PE concentrations were not significantly associated with EM risk (Fig. 2A).

To evaluate the impact of individual chemicals on EM, urinary EC concentrations were set at the 25th, 50th, or 75th percentiles. In the BKMR framework, the “univariate effect” represents the estimated exposure–response relationship for a single chemical when all other chemicals in the mixture are fixed at their median (50th percentile) concentrations. Among urinary heavy metals, Cs and Sb showed significant positive effects on EM. Among phthalates and PEs, MBP, ETD, and ETL were positively correlated with EM. For PAHs, 2-Flu was positively associated with EM, while 1-Pyr showed a negative correlation (Fig. 2B). Dose–response curves for 9 urinary heavy metals, 9 phthalates and PEs, and 8 PAHs in relation to EM are illustrated in Figure 2C.

### 3.5. WSR regression analysis

**Figure 3.**
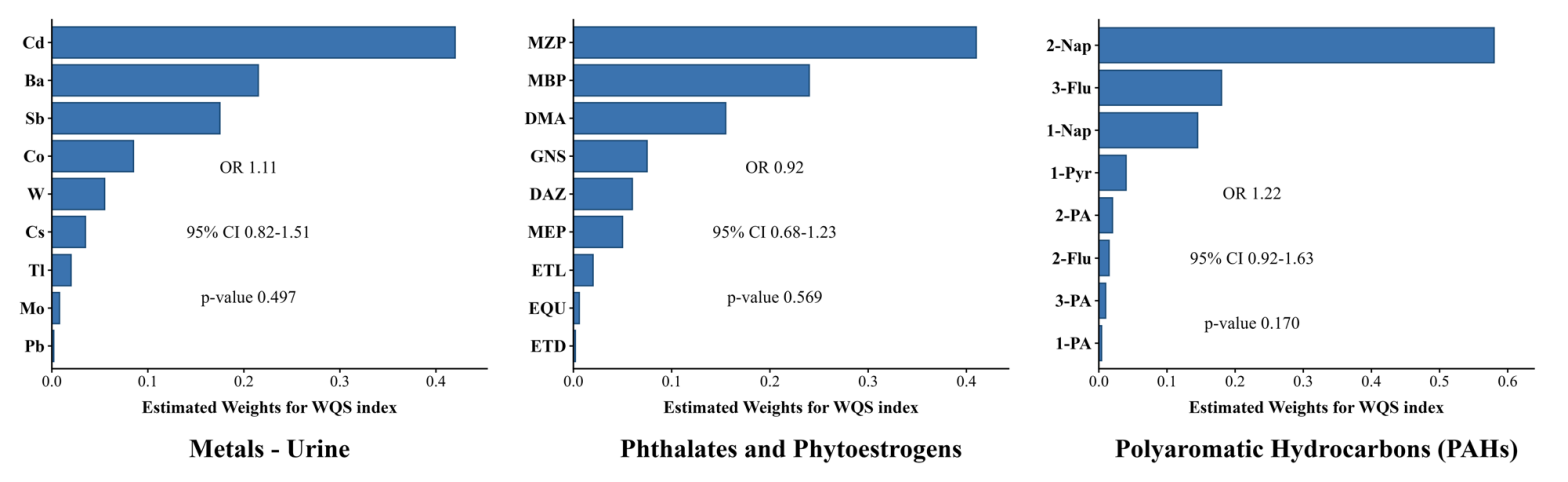
The weights of each chemical in WQS model regression index for EM. Adjusting by covariates such as age, ethnicity, education, marital status, family size, and household income. 1-Nap = 1-hydroxynaphthalene, 1-PA = 1-hydroxyphenanthrene, 1-Pyr = 1-hydroxypyrene, 2-Flu = 2-hydroxyfluorene, 2-Nap = 2-hydroxynaphthalene, 2-PA = 2-hydroxyphenanthrene, 3-Flu = 3-hydroxyfluorene, 3-PA = 3-hydroxyphenanthrene, Ba = barium, Cd = cadmium, Co = cobalt, Cs = cesium, DAZ = daidzein, DMA = O-desmethylangolensin, ECs = environmental chemicals, EM = endometriosis, EQU = equol, ETD = enterodiol, ETL = enterolactone, GNS = genistein, MBP = mono-n-butyl phthalate, MEP = monoethyl phthalate, Mo = molybdenum, MZP = monobenzyl phthalate, PAHs = polycyclic aromatic hydrocarbons, Pb = lead, Sb = antimony, Tl = thallium, W = tungsten, WQS = weighted quantile sum.

The WQS model was used to investigate the impact of mixed exposures on EM, focusing specifically on the association between urinary EC exposure and EM. Among urinary heavy metals, Cd had the highest weight (0.42), followed by Ba (0.21), Sb (0.17), and Co (0.08), while Pb had the lowest weight in the model. For phthalates and PEs, MZP had the highest weight (0.42), followed by MBP (0.41) and DMA (0.24), with ETD having the lowest weight. Among PAHs, 2-Nap had the highest weight (0.58), followed by 3-Flu (0.18) and 1-Nap (0.15), while 1-PA had the lowest weight. These findings indicated that Cd, MZP, and 2-Nap had the most significant impacts on EM. However, the WQS indices for urinary heavy metals, phthalates, PEs, and PAHs were not statistically significant in the analysis of their association with EM (Fig. 3).

## 4. Discussion

This study employed univariate logistic regression, WQS regression, and BKMR to examine the relationship between urinary ECs and EM. Specifically, the BKMR model was used to evaluate the overall impact of urinary heavy metals, phthalates, PEs, and PAHs on EM. The findings revealed that mixtures of urinary heavy metals and PAHs were positively associated with EM when their overall concentrations exceeded the 50th percentile. Notably, when urinary EC concentrations were fixed at different percentiles, Cs and Sb among the heavy metals showed significant positive effects on EM. Furthermore, MBP, ETD, ETL, and 2-Flu were positively correlated with EM, whereas 1-Pyr exhibited a negative correlation. In addition, WQS regression analysis indicated that Cd, MZP, and 2-Nap had more pronounced impacts on EM, although the WQS index itself was not significantly associated with the disease.

A noteworthy finding is the discrepant direction of association for 2-Flu between traditional logistic regression (inverse association in quartile analysis) and BKMR (positive association as part of the PAH mixture). Several factors may explain this contradiction: high collinearity among PAHs, nonlinearity/threshold effects, and effect modification within mixtures. A growing body of evidence suggests links between urinary endocrine disruptors and reproductive disorders, such as infertility and genitourinary tract abnormalities.^[20,21]^ In the context of EM, this study found no association between 7 phthalates and EM risk via logistic regression analysis. It is important to note that simultaneous exposure to multiple chemicals can complicate health outcome assessments, as shared exposure sources and metabolic pathways often lead to high covariance. Thus, when studying the relationship between urinary endocrine disruptors and EM,^[22,23]^ consideration of chemical mixtures is crucial.

The BKMR model assessed the overall association between urinary ECs and EM. Previous studies on the relationship between Pb and other heavy metal concentrations and EM have yielded inconsistent results: some indicate that Pb exposure increases EM risk,^[11]^ while others find no significant link. These discrepancies may stem from variations in study design, sample size, or exposure assessment methods. The current study suggests that higher Pb levels are associated with elevated EM risk, highlighting the need for further research into underlying biological mechanisms. PAHs – known carcinogens – have been extensively studied for their health impacts.^[15,16]^ In this study, PAHs such as 2-Flu were positively associated with EM risk, while 1-Pyr showed a negative correlation. PAHs may contribute to EM development by influencing cell proliferation and apoptosis.^[17,18]^ Toxicological studies have demonstrated that PAH exposure can cause DNA mutations and chronic inflammation in animals,^[24,25]^ potentially affecting gonadal maturation during puberty and exacerbating EM burden. The diverse exposure patterns and health effects of PAHs pose a significant public health challenge requiring ongoing attention.

MBP, ETD, and ETL among phthalates and PEs were positively correlated with EM. It is important to emphasize that the overall mixture of phthalates and PEs was not significantly associated with EM risk in the BKMR model. The positive signals observed for MBP, ETD, and ETL in the individual exposure–response plots should therefore be considered suggestive and model-dependent, requiring cautious interpretation and confirmation in future studies. Previous studies have also noted associations between PEs and EM,^[10]^ suggesting that PE exposure may impact the female reproductive system by influencing luteinizing hormone, follicle-stimulating hormone, gonadotropin-releasing hormone, and aromatase – potentially increasing EM prevalence.^[26]^ Given that EM is estrogen-dependent,^[27]^ EC exposure may elevate EM risk by disrupting these hormone regulators. Some research suggests PEs could contribute to EM development by influencing estrogen signaling pathways^[14]^; however, these findings require validation in larger population samples.

This study has several advantages over previous research. First, it uses data from the NHANES spanning 1999 to 2016 – a dataset recognized for its authority and accuracy – providing a robust foundation. Second, it employs diverse statistical methods, including univariate logistic regression, weighted logistic regression, BKMR, and WQS regression, ensuring a comprehensive assessment of associations between ECs and EM, with thorough and reliable results. Furthermore, its findings offer valuable insights for shaping public health policies.

This study also has limitations. First, the cross-sectional nature of NHANES data precludes establishing causality. Second, EC measurements may contain errors, and not all potential endocrine disruptors were evaluated. Third, certain variables (e.g., occupational data) were not included in analyses. Fourth, the database lacked data on some heavy metals, excluding potentially EM-relevant ones from the study. Fifth, EM was primarily defined via participant self-report, raising concerns that the control group may include undiagnosed cases – undermining the reliability of recall data. Lastly, not all potential confounders (e.g., lifestyle and genetic factors) were fully accounted for. Future research should use longitudinal designs to explore the temporal relationship between ECs and EM in depth, expand chemical testing, conduct more thorough confounder analyses, evaluate environmental interventions, and develop strategies for early identification and prevention in high-risk populations.

## 5. Conclusions

This study provides novel insights into the connection between EM and ECs. Results suggest a potential impact of heavy metals and PAH mixtures on EM development, while findings for phthalates and PEs remain tentative and model-dependent, requiring further investigation. The identified associations, particularly for cobalt, lead, and 2-Flu in mixture analysis, warrant deeper biological and epidemiological exploration.

## Acknowledgments

We thank all authors for their contributions to the article.

## Author contributions

**Methodology:** Yufeng Dong.

**Writing – original draft:** Yufeng Dong.

**Formal analysis:** Jing Qiu.

**Investigation:** Jing Qiu.

**Writing – review & editing:** Jing Qiu, Zhigang Tong.

**Resources:** Mengyun Yang.

**Software:** Mengyun Yang.

**Validation:** Wei Wang.

**Visualization:** Wei Wang.
